# Biomarkers of mitotoxicity after acute liver injury: Further insights into the interpretation of glutamate dehydrogenase

**Published:** 2021-01-27

**Authors:** Mitchell R. McGill, Hartmut Jaeschke

**Affiliations:** ^1^Department of Environmental and Occupational Health, Fay W. Boozman College of Public Health, University of Arkansas for Medical Sciences, 4301 W. Markham St, Little Rock, AR, 72205 USA; ^2^Department of Pharmacology and Toxicology, College of Medicine, University of Arkansas for Medical Sciences, 4301 W. Markham St., Little Rock, AR, 72205 USA; ^3^Department of Pharmacology, Toxicology and Therapeutics, University of Kansas Medical Center, 3901 Rainbow Blvd., Kansas City, KS, 66160 USA

**Keywords:** Acetaminophen, drug-induced liver injury, hepatotoxicity, mitochondria, translational research

## Abstract

**Background:**

Acetaminophen (APAP) is a popular analgesic, but overdose causes acute liver injury and sometimes death. Decades of research have revealed that mitochondrial damage is central in the mechanisms of toxicity in rodents, but we know much less about the role of mitochondria in humans. Due to the challenge of procuring liver tissue from APAP overdose patients, non-invasive mechanistic biomarkers are necessary to translate the mechanisms of APAP hepatotoxicity from rodents to patients. It was recently proposed that the mitochondrial matrix enzyme glutamate dehydrogenase (GLDH) can be measured in circulation as a biomarker of mitochondrial damage. Early observations revealed that damaged mitochondria release their contents into the cytosol. It follows that those mitochondrial molecules become freely detectable in blood after cell death. On the other hand, intact mitochondria would not release their matrix contents and can be removed from serum or plasma by high-speed centrifugation. However, a recent study cast doubt on the interpretation of GLDH as a mitotoxicity biomarker by demonstrating that neither high-speed centrifugation nor repeated freezing and thawing to lyse mitochondria alter GLDH activity in serum from mice with drug-induced liver injury.

**Aim:**

Here, we briefly review the evidence for mitochondrial damage in APAP hepatotoxicity and demonstrate that removal of intact mitochondria by centrifugation does not alter measured GLDH activity simply because GLDH within the mitochondrial matrix is not accessible for measurement. In addition, we show that freezing and thawing is insufficient for complete lysis of mitochondria.

**Relevance for Patients:**

Our literature review and data support the interpretation that circulating GLDH is a biomarker of mitochondrial damage. Such mechanistic biomarkers are important to translate preclinical research to patients.

Acetaminophen (APAP) is a widely used drug, but overdose causes severe centrilobular hepatocyte necrosis. It is currently the leading cause of acute liver failure in the US [1]. Decades of research using rodent models of APAP hepatotoxicity have indicated that mitochondrial damage and dysfunction are central in the molecular mechanisms of injury [2-4]. Ultrastructural evidence of mitochondrial damage was observed by electron microscopy in the 1980s [5]. Soon after, biochemical assays demonstrated loss of mitochondrial respiration [6] and development of mitochondrial oxidative stress [7]. The 1990s brought data on mitochondrial protein alkylation [8], and imaging studies in the 2000s revealed loss of mitochondrial membrane potential [9], which linked to peroxynitrite formation inside of the mitochondria [10]. Later, it was demonstrated that rats, which are less susceptible to APAP hepatotoxicity than mice, have much lower mitochondrial protein alkylation than mice even at higher doses [11]. Furthermore, an isomer of APAP, N-acetyl-*m*-aminophenol (AMAP), is less toxic in mice or mouse hepatocytes, correlating with the virtual absence of mitochondrial protein adducts after AMAP compared to APAP [12,13]. On the other hand, AMAP is more toxic to human hepatocytes than to mouse hepatocytes because the reactive metabolite of AMAP binds more to mitochondrial proteins in the human cells [13]. Those data demonstrate that mitochondrial protein binding is critical. More importantly, multiple interventions intended to either directly (cyclosporine A, Mito-Tempo, cyclophilin D KO) or indirectly (e.g., JNK inhibitors) reduce the mitochondrial dysfunction protect against APAP toxicity [9,14-20]. In addition, interventions designed to enhance the mitochondrial damage also increase injury [21]. Altogether, the data demonstrating that mitochondrial damage is necessary for APAP hepatotoxicity are clear and overwhelming.

Before the 2010s, little effort was made to translate the basic mechanisms of APAP hepatotoxicity identified in animal models to humans, beyond the early glutathione depletion and protein binding. We attempted to do that beginning in 2010 using samples from APAP overdose patients [22]. However, we faced a major challenge: We only had access to blood samples from these patients. In most cases, a liver biopsy is not necessary to make a diagnosis of APAP hepatotoxicity, so it is rare in the USA. We developed an alternative strategy: We used mouse models of centrilobular hepatocyte necrosis with and without mitochondrial dysfunction (APAP and furosemide [FS] overdose, respectively) and compared mitochondrial macromolecules in circulation to identify some that were specific for mitochondrial damage. Our hypothesis was that during significant mitochondrial damage, molecules from the mitochondrial matrix are released into the cytosol and then, following necrotic cell death, into the circulation. When we analyzed blood samples from mice, we found that mitochondrial DNA (mtDNA) and glutamate dehydrogenase (GLDH) were significantly elevated in plasma or serum from APAP-treated animals but not from FS-treated mice [22]. These data are consistent with the idea that mtDNA and GLDH can serve as mechanistic biomarkers for mitochondrial damage in patients with APAP hepatotoxicity. We then measured those biomarkers in plasma or serum from APAP overdose patients with liver injury and found that they were dramatically elevated in those patients compared to healthy volunteers [22]. On that basis, we concluded that mitochondrial damage does indeed occur in humans, similar to our observations in mice. We then confirmed these findings in a larger cohort of patients and found that higher serum levels of these biomarkers are modestly predictive of poor outcome, indicating that mitochondria could be a driver of the injury in humans [23]. In addition, other groups detected GLDH and mtDNA in serum from APAP overdose patients as well [24,25]. Finally, we found additional evidence of mitochondrial damage in cultured primary human hepatocytes [26] and in metabolically competent human HepaRG cells [27]. Importantly, mitochondrial dysfunction followed reactive metabolite formation and protein binding on mitochondria and preceded cell death in both primary human hepatocytes and the HepaRG cells [26,27].

In a recent article, Church *et al*. [28] concluded that GLDH is not a biomarker of mitochondrial damage after all. They drew that conclusion primarily from three major observations. First, GLDH was significantly elevated in serum from FS-treated mice in their hands. Second, GLDH activities correlated with ALT levels in serum from both APAP- and FS-treated mice. Third, and most importantly, neither centrifugation to pellet intact mitochondria nor repeated freezing and thawing to disrupt mitochondrial membranes affected GLDH activity in their samples [28]. However, although the authors did detect an increase in circulating GLDH activity in FS-treated mice in contrast to our earlier results, that increase was still much less than the elevation in their APAP-treated mice, with a GLDH/ALT ratio five-fold lower in the FS mice [28]. Overall, that is consistent with our fundamental observation that GLDH is lower in FS hepatotoxicity than in APAP hepatotoxicity. Furthermore, it is not surprising that GLDH correlates with ALT, since release of both ALT and GLDH would require cell necrosis and loss of plasma membrane integrity regardless of whether or not the mitochondria are still intact. Finally, the idea that high-speed centrifugation should reduce the measured GLDH activity if mitochondria are intact assumes that the GLDH assay can measure GLDH trapped within the mitochondrial matrix. In fact, the inner mitochondrial membrane tightly regulates movement of metabolic substrates in and out of the matrix, and one such substrate is a-ketoglutarate (aKG). Importantly, aKG is in most GLDH assay reagents (including the reagents used by Church *et al*.) and is required for measurement of GLDH activity. Thus, it is unlikely that the GLDH assay can detect matrix-localized GLDH in the first place. In addition, while repeated freezing and thawing is widely used to disrupt the plasma membrane of cells, it is less effective to disrupt sub-cellular organelles and the authors did not report that they verified breakage of the mitochondrial membranes in their experiments.

To determine if common GLDH assays can detect GLDH within the mitochondrial matrix and to test the effect of freezing and thawing, we performed a simple experiment with freshly isolated mitochondria that we obtained from the liver of an untreated wild-type mouse by differential centrifugation, as we previously described [29]. We re-suspended the isolated mitochondria in 1× phosphate-buffer saline (PBS) with 4 g/dL bovine serum albumin (BSA) to mimic serum, divided the suspension into six equal aliquots, and then mixed the aliquots 1:1 with PBS-BSA with or without 0.25% Triton X-100 to disrupt mitochondrial membranes (3 aliquots each). After incubating the samples on ice for 30 min, we centrifuged them at 20,000×g for 10 min at 4°C to pellet any remaining intact mitochondria and verified mitochondrial lysis in the detergent-treated samples by visual inspection. We then re-suspended the pellets again and measured GLDH in the suspensions using the same method that we use to measure GLDH in serum and plasma samples [22,23]. Importantly, incubation with detergent increased mean GLDH activity 332±30% (Figure 1), demonstrating that most GLDH within intact mitochondria cannot be measured using a standard GLDH assay. We then subjected these same samples to three freeze-thaw cycles and re-measured GLDH. While freezing and thawing further increased activity in the detergent-treated samples, the difference in values before and after freeze-thaw in the absence of detergent was not statistically significant (Figure 1) (*P* = 0.159; one-way analysis of variance (ANOVA) with post-hoc Holm-Sidak test for multiple comparisons). Together, these data indicate that the methods used by Church *et al*. [28] may not be ideal to test their hypothesis. In addition, our results indicate that specimen processing to remove intact mitochondria may not be necessary before freezing even when using GLDH as a biomarker of mitotoxicity, as we suggested previously [30].

**Figure 1:**
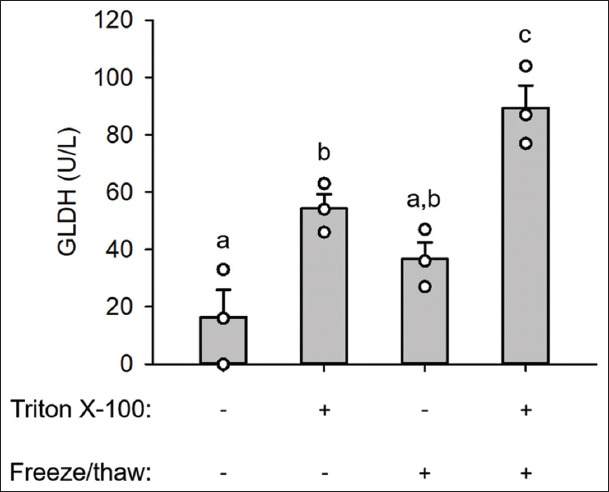
Glutamate dehydrogenase (GLDH) activity assays cannot measure GLDH within intact mitochondria. Mitochondria were freshly isolated by differential centrifugation from the combined right and caudate liver lobes of an untreated wild-type mouse. The mitochondria were then re-suspended in 1× phosphate buffered saline (PBS) containing 4 g/dL bovine serum albumin and divided equally into six aliquots. The aliquots were then mixed 1:1 with PBS+bovine serum albumin with (+) or without (−) 0.25% Triton X-100 and incubated on ice for 30 min. GLDH activity was then measured in each suspension. After the initial measurement, the samples were frozen and thawed 3 times and GLDH was re-measured. Data show mean±SE for n=3 aliquots. Dots show actual values. Bars with different letters are significantly different from one other based on one-way ANOVA with post-hoc Holm-Sidak test for multiple comparisons. P<0.05 is considered significant.

One caveat in our experimental design is that we used mitochondria isolated from the liver of a healthy mouse. It is possible that mitochondria from damaged tissue would yield somewhat different results. For example, stressed mitochondria may be more susceptible to lysis from freezing and thawing. In fact, the latter would be consistent with our data showing that freezing and thawing had a greater effect in the presence of detergent. On the other hand, the method of isolating mitochondria that we used is undoubtedly traumatic and may already stress the organelles to an extent that is similar to hepatotoxicity. Many additional experiments would be needed to test those possibilities in detail.

Although we disagree with their conclusion that GLDH is not a mitochondrial damage biomarker based on our data, the study by Church *et al*. [28] is nevertheless very interesting. Their observations that some mitochondria develop ultrastructural changes consistent with damage in FS hepatotoxicity and that mitophagy may be even more extensive in the FS model than in the APAP model indicate that mitophagy may be another reason for the lower GLDH release after FS treatment. Far from invalidating our earlier observations, we interpret these results as providing additional evidence that GLDH is in fact a biomarker of damaged mitochondria, especially when taken together with the results we have presented here. Essentially, their data indicate that having more damaged mitochondria within hepatocytes leads to greater serum GLDH, while having less (whether that is due to less initial mitochondrial damage or more efficient removal of damaged mitochondria) results in lower GLDH values. These important data demonstrate that we need to consider both the initial mitochondrial damage and later mitochondrial turnover when developing and characterizing mechanistic mitochondrial damage biomarkers. They also indicate that FS-induced hepatotoxicity in mice could be a useful model to study the role of mitophagy in drug-induced liver injury.

Together, our prior work and the results reported by Church *et al*. [28] indicate that the ratio of GLDH to ALT can provide insight into the role of mitochondrial damage in liver injury. We believe this can be useful in translational research when investigating mechanisms of disease, and in early drug development to test if mitochondria are potential therapeutic targets. In clinical practice and for regulatory purposes, GLDH may also be useful to distinguish liver from muscle as a source of elevated ALT in patients with muscle disease [31], though it is not yet clear how the combination of ALT and GLDH compares with the combination of ALT and creatine kinase that is already widely used in clinical laboratories for that purpose. Overall, we conclude that serum GLDH remains a useful biomarker of mitochondrial damage for translational studies of acute liver injury.
